# Severe traumatic valgus instability of the elbow: pathoanatomy and outcomes of primary operation

**DOI:** 10.1186/s13018-019-1374-8

**Published:** 2019-11-08

**Authors:** Lei Zhang, Laixu Wang, Shiyang Yu, Zhanhui Lv, Peng Zhang, Cunyi Fan, Yixin Shen

**Affiliations:** 1 Department of Orthopaedics, The Second Affiliated Hospital of Soochow University, 1055 Sanxiang Road, Suzhou, 215006 China; 2 Department of Orthopaedics, Binzhou Central Hospital Affiliated to Binzhou Medical University, 108 Huancheng South Road, Huimin, Binzhou, 251700 Shandong China; 3 Department of Orthopaedics, Shanghai Jiao Tong University Affiliated Sixth People’s Hospital, 600 Yishan Road, Shanghai, 200233 China

**Keywords:** Elbow, Medial collateral ligament, Radial head, Flexor-pronator tendon, Valgus, Instability

## Abstract

**Background:**

The objective of the study was to depict the pathoanatomy of traumatic valgus instability of the elbow and to report clinical outcomes of primary operation.

**Methods:**

Thirty-one patients presented with traumatic valgus instability of the elbow without dislocation. Thirty-one patients underwent surgical intervention of radial head fractures (28 open reduction and internal fixation and 3 radial head resection) and anatomical repair of the anterior bundle of medial collateral ligament (AMCL) with suture anchors. Twenty patients with disruption of the flexor-pronator tendon (FPT) and 14 patients with tears of the anterior capsule had primary repair of the FPT and anterior capsule simultaneously. Clinical outcomes were evaluated with the Mayo Elbow Performance Score (MEPS), modified hospital for special surgery assessment scale (HSS), and Disabilities of the Arm, Shoulder, and Hand (DASH) score.

**Results:**

The median follow-up was 37.3 months (range, 15–53 months). Radial head fractures and complete avulsion of the medial collateral ligament (MCL) from its humeral footprint were confirmed in all patients intraoperatively. Intraoperative findings indicated disruption of the FPT in 20 patients and tears of the anterior capsule in 14 patients. Twenty-nine of 31 patients returned to previous activity and work levels within 6 months after surgery. The MEPS, modified HSS, and DASH score were 94 ± 4, 91 ± 5, and 8 ± 2 at the latest follow-up.

**Conclusions:**

Radial head fractures with avulsion of the MCL can lead to severe valgus instability of the elbow. Primary operation to repair these disrupted structures, especially repair of the AMCL, can effectively restore valgus stability.

## Background

The native stability of the elbow depends on the integrity and coordination of the bone structures and the soft tissue structures [1–4]. The medial collateral ligament (MCL), specifically the anterior bundle of MCL (AMCL), functions as the primary static stabilizer to valgus stress, versus the radial head functions as the secondary static stabilizer [1, 5]. Lately, the importance of the flexor-pronator tendon (FPT) in maintaining valgus stability has attracted more and more attention [6–10]. Although the function of the FPT is not yet explicit, it may be a vital dynamic constraint to valgus stress, and its disruption can aggravate valgus instability of the elbow. Previous studies have shown that the joint capsule, specifically the anterior joint capsule, contributes critical resistance to valgus stress, joint hyperextension, and distraction [1]. In conclusion, valgus stability of the elbow is closely related to the integrity of the AMCL, the radial head, the FPT, and the anterior joint capsule.

The pathophysiology, clinical features, and treatment of valgus instability resulting from ligamentous injuries in dislocation of the elbow have been well studied [7, 11, 12]. A few studies have also reported on acute valgus instability resulting from tears of medial stabilizers without elbow dislocation [10, 13, 14]. Severe traumatic valgus instability may be associated with the rupture of the MCL and lesions of the radiocapitellar articulation. Davidson et al. [15] reported varying degrees of acute valgus instability of the elbow due to avulsion of the MCL in 22 of 50 cases of radial head fractures. Itamura et al. [16] evaluated the incidence of ligament injuries in radial head fractures by magnetic resonance imaging (MRI) and found disruption of the MCL in 54% of cases. Wapler et al. [17] reported tears of the MCL in 23 of 33 cases of radial head fractures (Mason type II and III). A few biomechanical experiments have studied the influence on valgus laxity of radial head fracture in the MCL-deficient elbow and the optional treatments of this instability pattern [5, 18, 19]. The traumatic mechanism, pathoanatomy, and treatment maneuvers of acute gross valgus instability of the elbow have been not well researched.

The objective of our study was to probe into the pathoanatomy of severe traumatic valgus instability of the elbow without dislocation based upon physical examination, imaging data, and operative findings and to assess clinical effects of primary operation.

## Materials and methods

**Fig. 1:**
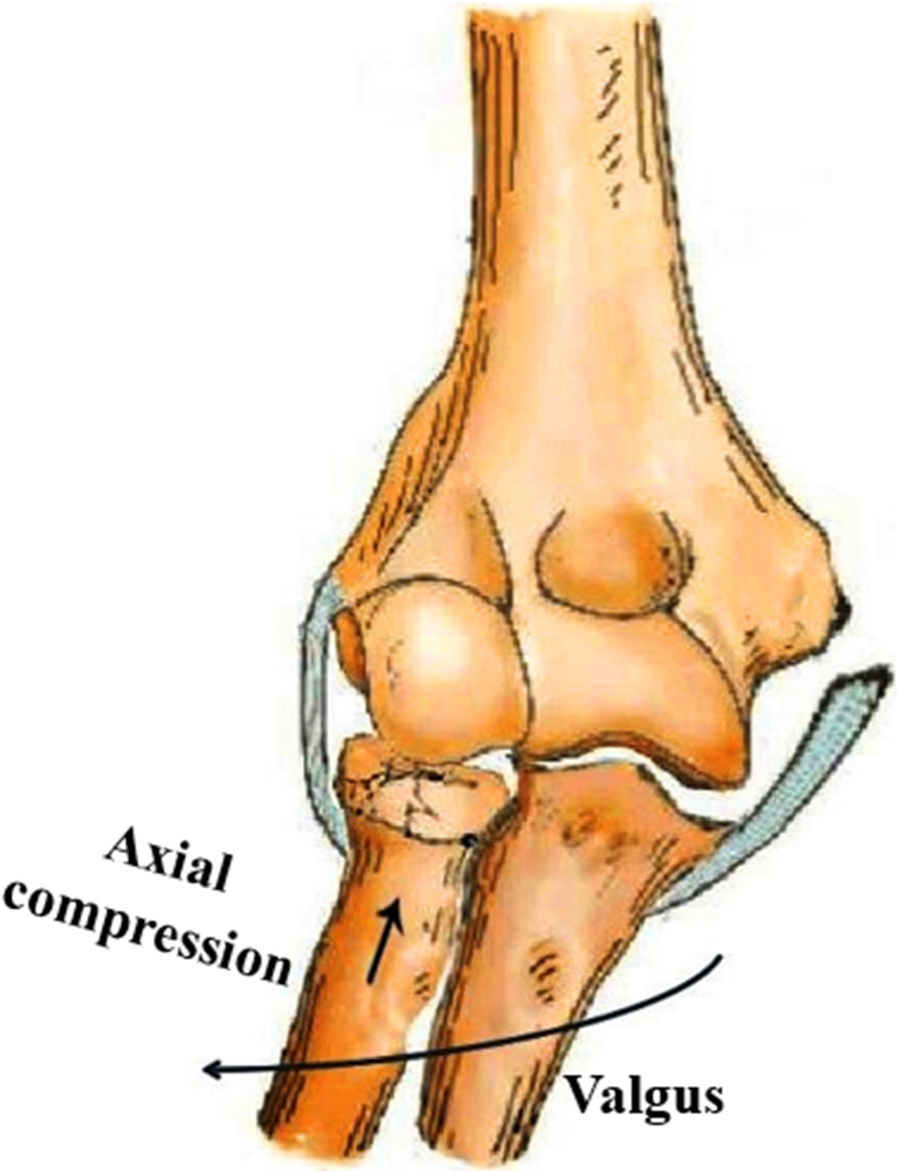
Radial head fractures with avulsion of the medial collateral ligament leading to gross valgus instability of the elbow

This was a retrospective case series study of patients with traumatic gross valgus instability of the elbow in our institution between April 2007 and October 2016. During the period, 35 patients with radial head fractures and avulsion of the MCL were included in this study. All patients had no history of elbow dislocation. Patients with chronic valgus instability during 3 weeks after an initial injury, radial head fractures combined with distal radioulnar joint dislocation, or associated fractures, including distal humerus, olecranon, or coronoid, were excluded from this study. Four of the 35 patients were lost to follow-up. The remaining 31 patients included 21 males and 10 females with an average age of 41.8 years (range, 18–69 years). All patients suffered from acute unilateral closed injury, which involved the dominant arm in 14 patients and the nondominant arm in 17 patients. The Mason-Hotchkiss classification was used to categorize radial head fractures [20]. Elbow radiographs demonstrated radial head fractures were Mason type II in 22 elbows, type III in 9. The mechanism of trauma was a fall on an outstretched hand with a serious valgus strain force on the elbow for 27 patients (fall from high places, 4; ground-level fall, 5; sports activities, 8; tripped, 10) (Fig. 1). Four patients were injured in traffic accidents, but they could not recall the specific position of the elbow at the time of trauma. The median follow-up time was 37.3 months (range, 15–53 months) (Table 1).

**Table 1:** Thirty-one patients’ clinical data

Case no.	Sex	Age (years)	Dominance	Span from trauma to operation (days)	Difference in MJA (VSR, degrees)	Final MEPS	Final modified HSS	Final DASH	Follow-up (months)	Complications
1	M	21	+	5	9.5	97	94	7	44	
2	M	52	–	7	9	95	93	9	53	
3	F	18	+	4	9.8	100	100	5	35	
4	M	31	+	3	8	100	100	5	28	
5	M	43	–	6	10	94	90	9	48	
6	M	28	–	8	11	93	91	9	49	
7	M	51	+	5	8.5	96	93	8	51	
8	M	51	–	9	9.5	90	86	9	36	
9	F	36	+	4	7.9	99	98	7	24	
10	F	23	–	8	10.5	95	89	9	37	
11	M	54	–	11	13.8	86	82	13	47	HO
12	M	41	–	6	9.1	94	90	8	19	
13	M	37	+	4	8	99	94	6	43	
14	F	49	–	8	9.1	92	89	8	48	
15	M	54	+	10	10.8	92	87	10	26	HO
16	F	50	–	8	9.2	89	85	9	30	
17	M	38	+	6	7.8	98	93	6	35	
18	M	48	+	10	12.4	89	85	10	53	
19	M	51	–	8	9.4	93	90	8	15	
20	F	40	–	6	9	96	94	7	25	
21	M	49	–	10	12.5	88	85	11	46	HO
22	M	35	+	5	8.4	99	97	6	22	
23	F	50	–	9	11.5	89	82	10	40	
24	M	26	+	5	8.5	99	97	6	45	
25	F	39	+	6	9.3	91	89	8	50	
26	M	47	–	7	8.8	95	92	7	32	
27	F	69	–	11	14.6	85	82	13	26	
28	M	41	+	10	13	87	84	12	42	HO
29	F	51	–	9	9	91	87	9	33	
30	M	25	+	5	8.8	98	96	6	30	
31	M	49	–	6	8.6	97	93	7	45	

*DASH* Disabilities of the Arm, Shoulder, and Hand score, *F* female, *HO* heterotopic ossification, *HSS* hospital for special surgery assessment scale, *M* male, *MEPS* Mayo Elbow Performance Score, *MJA* medial joint angulation, *VSR* valgus stress radiograph

The symptoms of swelling and pain on the medial and lateral sides of the elbow, especially the medial side, were evaluated in all patients. Clinical examination showed widespread subcutaneous hematomas involving the medial side of the elbow joint, the proximal forearm, and the distal humerus. Valgus stress test under general anesthesia and fluoroscopy was performed with the upper arm fixed and the elbow in 30° of flexion, where the olecranon was unlocked from the olecranon fossa to facilitate the evaluation of valgus stability. The contralateral elbow was also examined for comparison. All elbows demonstrated remarkable valgus instability without a fixed endpoint. Two patients had the symptoms of ulnar nerve and posterior interosseous nerve (PIN) acute incomplete paralysis, and 3 patients had only ulnar nerve incomplete paralysis at the time of the trauma.

**Fig. 2:**
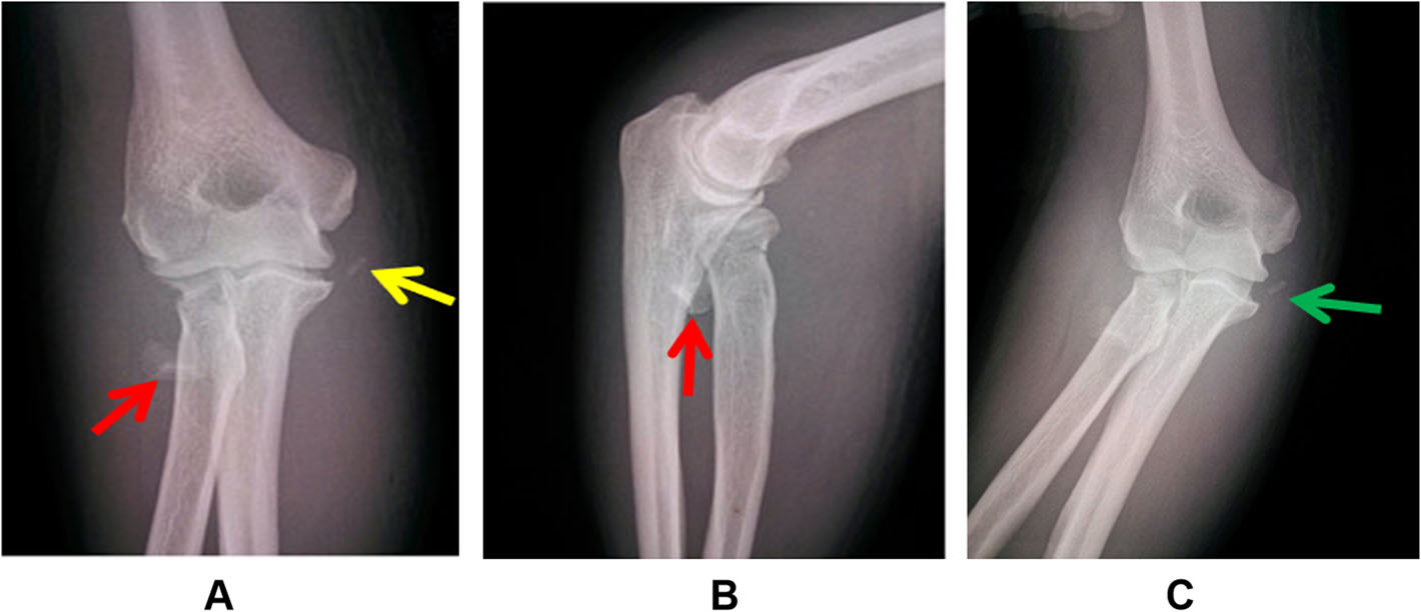
Anteroposterior (**a**) and lateral (**b**) radiographs of the injured elbow. The fracture fragment of the radial head (red arrow) is remarkably displaced. A free bone fragment is found on the medial side of the elbow (yellow arrow). The valgus stress radiograph of the injured elbow (**c**) demonstrates medial joint space widening (green arrow)

**Fig. 3:**
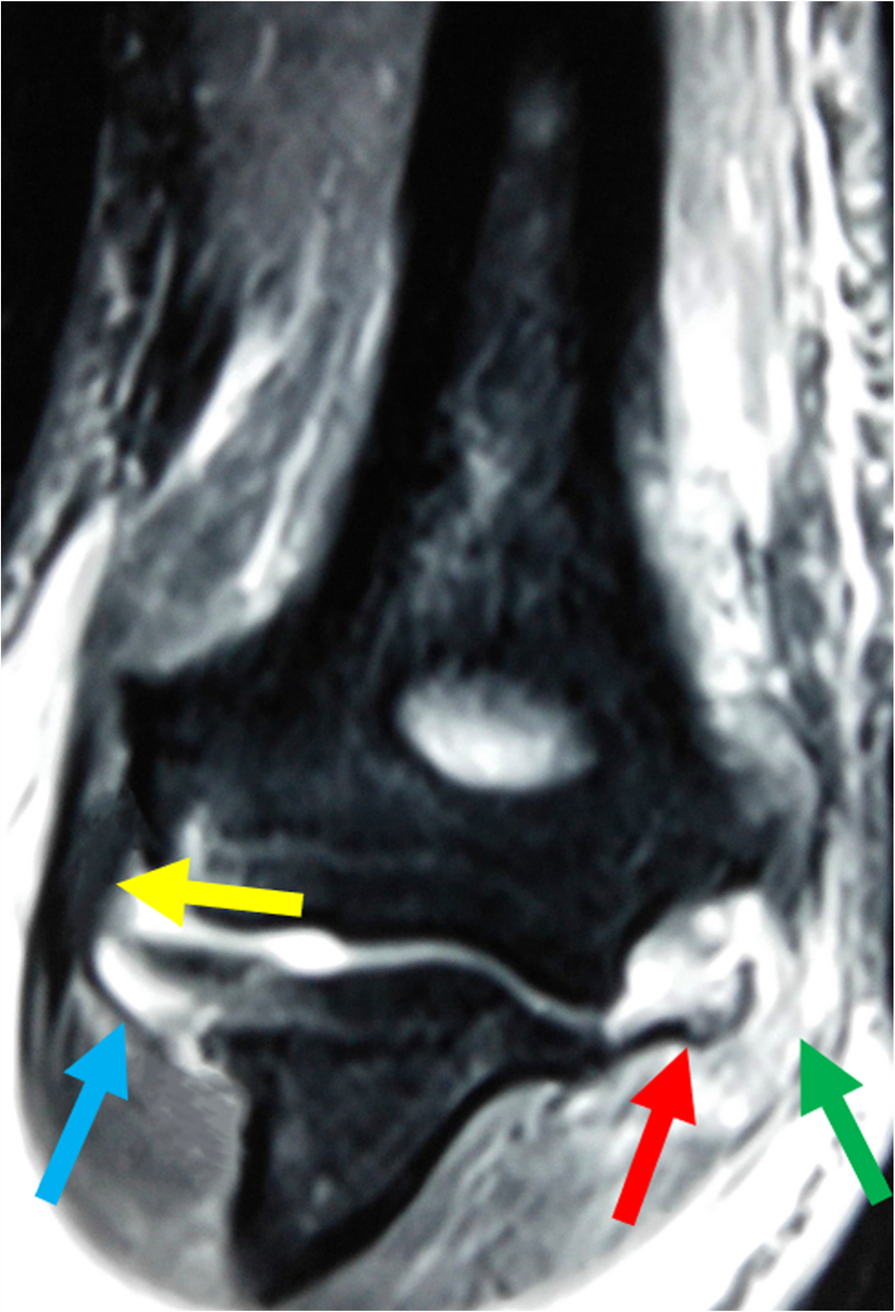
T2-weighted coronal image from magnetic resonance imaging. The medial collateral ligament (red arrow) is avulsed from its humeral footprint. The flexor-pronator tendon is torn as well (green arrow). There is an increased signal in their origin on the medial epicondyle. The lateral collateral ligament (yellow arrow) and the lateral ulnar collateral ligament (blue arrow) are intact

Imaging studies included plain (anteroposterior and lateral) radiographs, valgus stress radiographs (Fig. 2), and standard MRI (Fig. 3). All patients underwent plain radiographs to assess radial head fractures. Valgus stress radiographs were used to assess the stability of all elbows. The medial joint angulation between the distal humeral joint line and the proximal ulno-radial joint line was measured in maximal valgus stress [21], which was compared with the contralateral elbow. The average difference in the medial joint angulation was 9.8° (range, 7.5–14.6°), which indicated gross valgus instability of the elbow. All elbows were evaluated by an experienced musculoskeletal radiologist using standard MRI, which showed complete avulsion of the MCL from its humeral footprint and marrow edema of the humeroradial joint. No pathology of the lateral collateral ligament complex (LCLC) was detected.

**Fig. 4:**
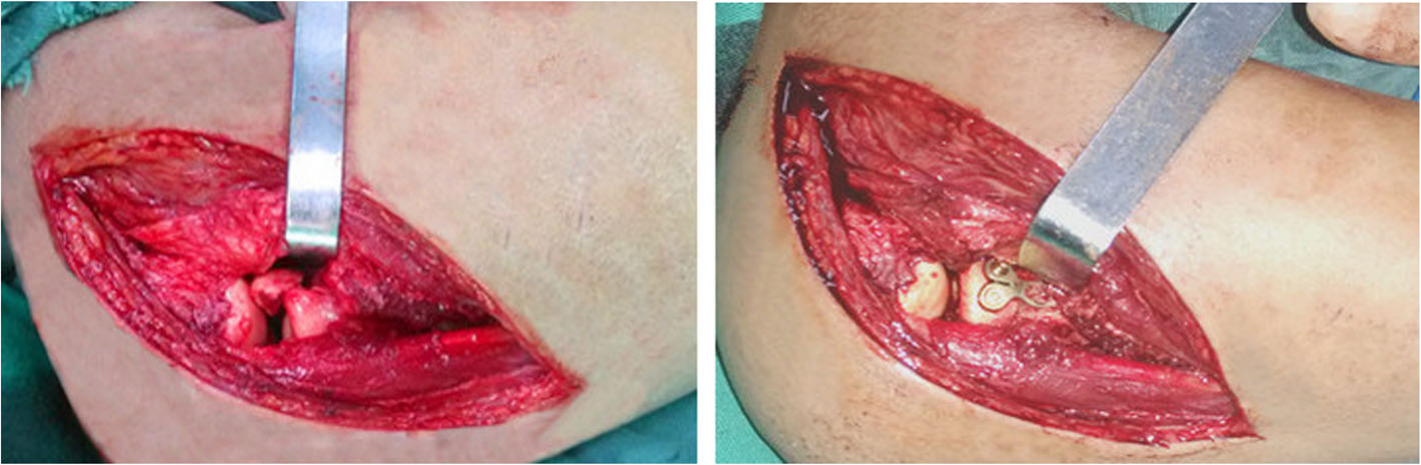
Intraoperative photographs show anatomical reduction and internal fixation of the type II radial head fracture

**Fig. 5:**
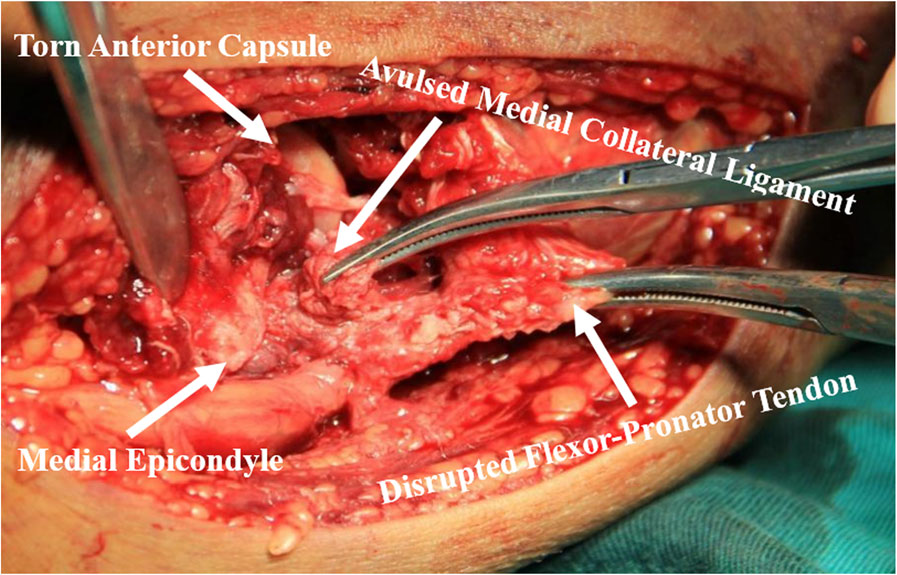
Intraoperative photograph shows the denuded medial epicondyle, the torn anterior capsule, and the avulsed medial collateral ligament and flexor-pronator tendon. The ulnohumeral joint is visualized deep to the injured structures

The mean time span from trauma to operation was 7.1 days (range, 3–11 days). Valgus instability of the elbow joint was confirmed under general anesthesia. Firstly, the Kocher approach was utilized for exposing and treating radial head fractures (Fig. 4) and exploring the conditions of PIN and the LCLC simultaneously. Anatomical reduction and internal fixation were then performed for radial head fractures in 28 patients, of which 16 cases were fixed with headless compression screws (Φ3.0 mm) and 12 cases were fixed with T-mini-plates. Resection of the radial head was performed for the remaining 3 patients with comminuted and unreconstructable fractures. Valgus instability of the elbow on intraoperative lateral stress test under fluoroscopy was reconfirmed after addressing radial head fractures and closing the Kocher incision, and then, the medial approach (Hotchkiss) was utilized for exposing and evaluating the conditions of FPT, MCL, joint capsules, and ulnar nerve (Fig. 5).

All patients with complete humeral-sided avulsion of the MCL had anatomical repair of the AMCL with suture anchors screwed into its footprint on the medial epicondyle with appropriate ligamentous tension as the elbow was held in slight varus and 45° of flexion. Twenty patients with disruption of the FPT and 14 patients with tears of the anterior joint capsule underwent primary repair of the FPT and anterior joint capsule simultaneously. The FPT was repaired back to its origin of the medial epicondyle with suture anchors in 14 of the 20 patients. In the remaining 6 patients, the disrupted FPT was repaired back to the musculotendinous junction with no. 2 nonabsorbable sutures. The torn anterior capsule was repaired with interrupted nonabsorbable sutures. Hematoma was detected in the cubital tunnel, and the ulnar nerve was edematous and hemorrhagic in 5 patients with symptoms of ulnar nerve paralysis. The ulnar nerve was transposed anteriorly with the use of a fascial sling to avoid being compressed by the hematoma in the cubital tunnel in these 5 patients.

All elbows were placed in a hinged brace locked at 90° with the forearm in neutral rotation for 2 weeks postoperatively. At 2 weeks after the operation, the brace was unlocked, and early passive and subsequent active elbow exercises were allowed. All patients engaged in muscle strengthening exercises after the brace was taken off at 8 weeks, and the instruction for avoiding valgus stress for at least 12 weeks was given. They were encouraged to engage in a previous activity and work by 14 or 16 weeks after operation.

Clinical outcomes were evaluated with the Mayo Elbow Performance Score (MEPS), the modified hospital for special surgery assessment scale (HSS), and the Disabilities of the Arm, Shoulder, and Hand (DASH) score [22]. Complications such as instability of the elbow, nerve damage, articular degeneration, heterotopic ossification (HO), and stiffness were also assessed.

## Results

Intraoperative findings confirmed radial head fractures were Mason type II in 22 elbows, type III in 9. The fracture block was found to compress PIN in 2 patients. No abnormalities of LCLC were founded in all patients. Bone contusion at the capitellum was found in 28 patients. Complete avulsion of the MCL in a periosteal sleeve pattern from the humeral footprint was found in all patients, and the torn end of ligament was folded into the ulnohumeral joint in 17 patients. The FPT was disrupted at the musculotendinous junction in 6 patients and at the origin of the medial epicondyle in 14 patients. Of the 14 patients, 9 patients sustained complete tears of the anterior capsule simultaneously and 5 patients sustained partial tears. Hematoma was detected on the floor of the cubital tunnel in 5 patients with symptoms of ulnar nerve paralysis (Table 2).

**Table 2:** Pathoanatomy of severe traumatic valgus instability based on radiographs, MRI, and intraoperative findings

Case no.	Mason classification	MCL	FPT	LCLC	Capitellum	Anterior capsule	UN	PIN
1	II	T(H)	T(H)	I	C	P	I	I
2	II	T(H)	I	I	C	I	I	I
3	II	T(H)	D	I	C	I	I	I
4	II	T(H)	I	I	I	I	I	I
5	II	T(H)	T(H)	I	C	P	I	I
6	III	T(H)	T(H)	I	C	T	IP	I
7	II	T(H)	I	I	C	I	I	I
8	II	T(H)	T(H)	I	C	P	I	I
9	II	T(H)	I	I	C	I	I	I
10	III	T(H)	T(H)	I	C	T	I	I
11	III	T(H)	T(H)	I	C	T	IP	IP
12	II	T(H)	D	I	C	I	I	I
13	II	T(H)	I	I	I	I	I	I
14	II	T(H)	D	I	C	I	I	I
15	III	T(H)	T(H)	I	C	T	I	I
16	II	T(H)	D	I	C	I	I	I
17	II	T(H)	I	I	C	I	I	I
18	III	T(H)	T(H)	I	C	T	IP	I
19	II	T(H)	I	I	C	I	I	I
20	II	T(H)	I	I	I	I	I	I
21	III	T(H)	T(H)	I	C	T	IP	I
22	II	T(H)	I	I	C	I	I	I
23	III	T(H)	T(H)	I	C	T	I	I
24	II	T(H)	D	I	C	I	I	I
25	II	T(H)	T(H)	I	C	P	I	I
26	II	T(H)	I	I	C	I	I	I
27	III	T(H)	T(H)	I	C	T	I	I
28	III	T(H)	T(H)	I	C	T	IP	IP
29	II	T(H)	D	I	C	I	I	I
30	II	T(H)	T(H)	I	C	P	I	I
31	II	T(H)	I	I	C	I	I	I

*C* contusion, *D* disruption at the musculotendinous junction, *FPT* flexor-pronator tendon, *H* humeral footprint, *I* intact, *IP* incomplete paralysis, *LCLC* lateral collateral ligament complex, *MCL* medial collateral ligament, *P* partial tear, *PIN* posterior interosseous nerve, *T* total tear, *UN* ulnar nerve

At the latest follow-up, the mean MEPS was 94 (range, 85–100), the mean modified HSS was 91 (range, 82–100), and the mean DASH score was 8 (range, 5–13). The mean flexion of the elbow was 128° (range, 110–140°), and the mean extension lag of the elbow was 5.7° (range, 0–15°). The symptoms of acute ulnar nerve paralysis in 5 patients and of PIN paralysis in 2 patients at the time of the trauma vanished within 5 months. HO around radial head occurred in 1 patient, around the medial epicondyle in 2 patients, and around both radial head and the medial epicondyle in 1 patient. Three of the 4 patients had satisfactory ranges of motion, and no pain. The remaining patient had a 25° flexion contracture with moderate pain, and the symptoms were relieved by physical therapy combined with taking NSAID drugs.

All elbows had no valgus instability or pain with valgus stress test on clinical examination at the latest follow-up. Twenty-nine of all 31 patients returned to previous activity and work levels within 6 months. Of the remaining 2 patients, one patient aged 69 years old had a poor treatment compliance and did not return to previous activity level. The other one with HO around both radial head and the medial epicondyle had a 25° flexion contracture, but he returned to a similar activity level as before the trauma after systematic physical therapy and rehabilitation exercise.

## Discussion

This clinical study demonstrated that radial head fractures combined with complete avulsion of the MCL can result in severe traumatic valgus instability of the elbow. Primary operation of these disrupted stabilizers can effectively restore valgus stability with excellent clinical efficacies.

The pathoanatomy and treatment maneuvers of chronic valgus instability of the elbow due to iterative microtrauma following overhead throwing activities have been well researched [23–27]. Nevertheless, acute valgus instability of the elbow sustains a different traumatic mechanism and so leads to a different pathoanatomy [10]. Schwab et al. [28] hypothesized the reasons why MCL tears often occur at the same time as the radial head fractures. An axial load with a serious valgus strain force on the elbow is likely to result in radial head fractures accompanied with MCL avulsion, which gives rise to acute gross valgus laxity. The suitable treatment maneuvers should be undertaken according to traumatic mechanism as well as pathoanatomy to regain valgus stability and function of the elbow.

Richard et al. [14] reported complete avulsion of the MCL from its humeral footprint and disruption of the FPT leading to acute traumatic valgus instability in 11 collegiate athletes. Cho et al. [10] reported 7 patients with acute gross valgus instability without elbow dislocation suffered from complete disruption of the MCL and FPT, with variable degrees of tears of the anterior capsule and bone contusion of the radial head and capitellum. Those authors believed that acute valgus instability is probably the initial phase of posterolateral dislocation of the elbow [10]. Davidson et al. [15] reported varying degrees of acute valgus instability of the elbow due to avulsion of the MCL in 22 of 50 cases of radial head fractures. In terms of traumatic mechanism and pathoanatomy, our results are extremely similar to those clinical studies mentioned above. In this study, all patients suffered from radial head fractures and complete avulsion of the MCL from the humeral footprint, of which 64.5% (20/31) were accompanied by disruption of the FPT and 45.2% (14/31) were accompanied by tears of the anterior capsule. We believe that the deforming forces consisting of valgus strain and axial compression to an extended elbow may lead to a sequence of disruption of the stabilizing structures from medial to lateral, thereby facilitating traumatic valgus instability. The average difference in the medial joint angulation was 9.8° on valgus stress radiographs when the injured elbow was compared with the contralateral elbow, which met the criterion of gross valgus instability [29].

Although the current treatment strategy of acute traumatic valgus instability has been still in controversy, most literature advocated primary repair of the anatomical stabilizers maintaining valgus stability of the elbow [10, 13, 14, 30]. Richard et al. [14] concluded that direct repair of an acute traumatic avulsion of the MCL utilizing the bone tunnel repair technique in 9 elbows and the suture anchor repair technique in 2 elbows regains valgus stability effectively. Cho et al. [10] believed that the operative indication for direct repair of acute valgus laxity of the elbow is severe valgus instability on stress testing and complete tears of the MCL and FPT on MRI, and concluded that primary repair of these medial structures with suture anchors is effective in avoiding late ligaments reconstruction, restoring stability and preventing poor outcomes. Although not explicitly mentioned in the above reports, valgus stability and function were regained effectually only by repairing the AMCL in the elbow with avulsion of the MCL.

There were few reports on the biomechanical experiments concerning the optional treatments of valgus instability resulting from radial head fractures and division of the MCL. Charalambous et al. [5] made a human cadaveric model comparing fracture fixation, excision, and radial head replacement in the elbow with two-part radial head fracture and division of the AMCL, and concluded that fixation has a biomechanical advantage over excision and replacement in terms of valgus and varus stability. However, those authors did not perform a primary repair of the disrupted AMCL, nor did they make relative biomechanical comparisons. Jensen et al. [18] researched the stabilizing effect of repair of MCL and prosthetic replacement of the radial head in five cadaver elbows with division of the MCL and excision of the radial head. Their results manifested that direct repair of the AMCL alone assuredly regains stability after division of the MCL. They concluded that the radial head is a constraint secondary to the MCL for valgus displacement; repair of the AMCL alone is sufficient to regain valgus stability in the elbow with disruption of the MCL and unreconstructable fractures of the radial head [18]. Our clinical study confirmed this viewpoint.

**Fig. 6:**
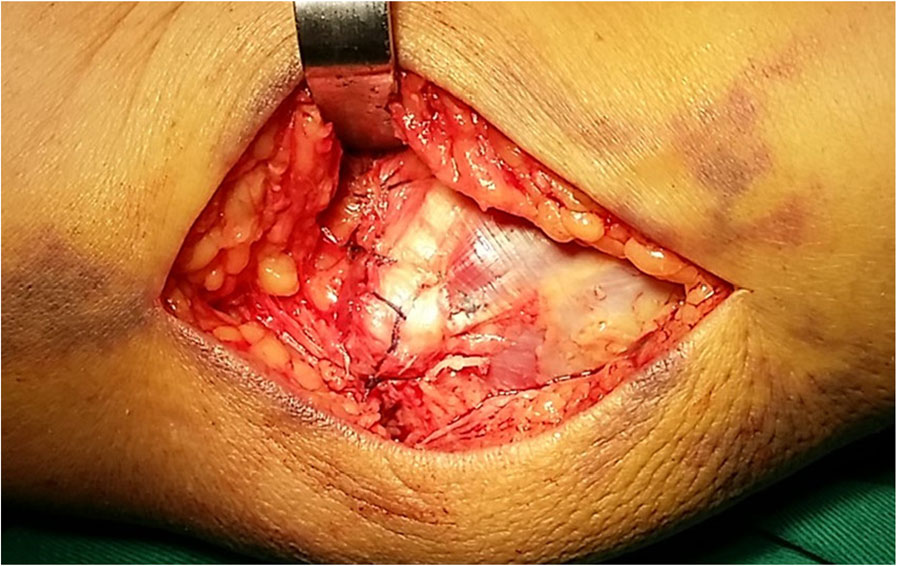
The medial stabilizers are repaired with suture anchor and nonabsorbable sutures

In the present study, all 31 patients sustained gross traumatic valgus instability resulting from radial head fractures and disruption of the medial stabilizers of the elbow. Open reduction and internal fixation were performed for radial head fractures in 28 patients, and resection of radial head was performed for 3 patients with comminuted and unreconstructable fractures. Elbow instability on intraoperative valgus stress test under fluoroscopy was reconfirmed after the lateral approach was completed in all patients, which confirmed once more that the radial head is a stabilizer secondary to the MCL for valgus stress [1, 5, 18], and treating radial head fractures alone is not enough to restore valgus stability in the presence of deficiency of the medial stabilizers, especially the MCL. Hence, all patients had primary repair of AMCL with suture anchors. Twenty patients with disruption of the FPT and 14 patients with tears of the anterior joint capsule underwent anatomical repair of the FPT and anterior joint capsule simultaneously. Primary repair of these stabilizers (Fig. 6) regained valgus stability in all patients and led to a functional restoration to the previous activity and work levels in 29 of all 31 patients at the latest follow-up.

There were several limitations in this study. First of all, the number of cases was relatively small and all patients suffered from severe traumatic valgus instability of the elbow and underwent surgical treatment. We did not set a control group with conservative treatment. Although the surgical treatment of this trauma had regained valgus stability and achieved satisfactory clinical results, it is not clear whether surgical treatment has an advantage over conservative treatment. Second, the follow-up time was relatively short. While we observed excellent outcomes at the last follow-up, a longer follow-up is required to evaluate the late complications such as articular degeneration and heterotopic ossification. Also, we did not perform a biomechanical experiment on traumatic valgus instability resulting from radial head fracture and division of the MCL, which is likely to make important contributions to our clinical study in terms of precise traumatic mechanisms, classification for valgus severity, surgical indications, and repair techniques.

## Conclusions

This clinical study demonstrated that radial head fractures combined with complete avulsion of the MCL can result in severe traumatic valgus instability of the elbow. Primary operation to repair these disrupted structures, especially repair of the AMCL, can effectively restore valgus stability.

## Data Availability

The datasets used and/or analyzed during the current study are available from the corresponding author on reasonable request.

## Abbreviations

AMCL: Anterior bundle of medial collateral ligament
DASH: Disabilities of the Arm, Shoulder, and Hand
FPT: Flexor-pronator tendon
HO: Heterotopic ossification
HSS: Hospital for special surgery assessment scale
LCLC: Lateral collateral ligament complex
MCL: Medial collateral ligament
MEPS: Mayo Elbow Performance Score
MRI: Magnetic resonance imaging
PIN: Posterior interosseous nerve
